# Population cycles and outbreaks of small rodents: ten essential questions we still need to solve

**DOI:** 10.1007/s00442-020-04810-w

**Published:** 2020-12-28

**Authors:** Harry P. Andreassen, Janne Sundell, Fraucke Ecke, Stefan Halle, Marko Haapakoski, Heikki Henttonen, Otso Huitu, Jens Jacob, Kaja Johnsen, Esa Koskela, Juan Jose Luque-Larena, Nicolas Lecomte, Herwig Leirs, Joachim Mariën, Magne Neby, Osmo Rätti, Thorbjörn Sievert, Grant R. Singleton, Joannes van Cann, Bram Vanden Broecke, Hannu Ylönen

**Affiliations:** 1 Faculty of Applied Ecology, Agricultural Sciences and Biotechnology, Inland Norway University of Applied Sciences, Campus Evenstad, 2480 Koppang, Norway; 2 Lammi Biological Station, University of Helsinki, Pääjärventie 320, 16900 Lammi, Finland; 3 Department of Biological and Environmental Science, Konnevesi Research Station, University of Jyväskylä, P.O. Box 35, 40014 Jyväskylä, Finland; 4 Department of Wildlife, Fish, and Environmental Studies, Swedish University of Agricultural Sciences, Skogsmarksgränd, 90183 Umeå, Sweden; 5 Institute of Ecology and Evolution, Friedrich Schiller University Jena, Dornburger Str. 159, 07743 Jena, Germany; 6 Terrestrial Population Dynamics, Natural Resources Institute Finland, Latokartanonkaari 9, 00790 Helsinki, Finland; 7 Federal Research Centre for Cultivated Plants, Vertebrate Research, Julius Kühn-Institut, Toppheideweg 88, 48161 Münster, Germany; 8 Department of Biological and Environmental Science, University of Jyväskylä, P.O. Box 35, 40014 Jyväskylä, Finland; 9 Departamento de Ciencias Agroforestales, Escuela Tecnica Superior de Ingenierıas Agrarias, Universidad de Valladolid, Campus La Yutera, Avenida de Madrid 44, 34004 Palencia, Spain; 10 Canada Research Chair in Polar and Boreal Ecology and Centre D’Études Nordiques, Department of Biology, Université de Moncton, 18 Avenue Antonine-Maillet, Moncton, NB E1A 3E9 Canada; 11 Evolutionary Ecology Group, Department of Biology, University of Antwerp, Universiteitslain 1, 2610 Wilrijk, Belgium; 12 Arctic Centre, University of Lapland, P.O. Box 122, 96101 Rovaniemi, Finland; 13 International Rice Research Institute, DAPO Box 7777, Metro Manila, Philippines; 14 Natural Resources Institute, University of Greenwich, Chatham Marine, Kent, ME4 4TB UK

**Keywords:** Density dependence, Phase dependence, Voles, Mice, Lemmings

## Abstract

Most small rodent populations in the world have fascinating population dynamics. In the northern hemisphere, voles and lemmings tend to show population cycles with regular fluctuations in numbers. In the southern hemisphere, small rodents tend to have large amplitude outbreaks with less regular intervals. In the light of vast research and debate over almost a century, we here discuss the driving forces of these different rodent population dynamics. We highlight ten questions directly related to the various characteristics of relevant populations and ecosystems that still need to be answered. This overview is not intended as a complete list of questions but rather focuses on the most important issues that are essential for understanding the generality of small rodent population dynamics.

## Introduction

Populations of small rodents have fascinated ecologists all over the world due to their extreme eruptive dynamics, or regular periodic fluctuations known as multiannual population cycles. Population cycles have fuelled decades of research since Charles Elton (1924, 1942), who described this phenomenon based on historical data in northwest Europe and Canada (Lindström et al. 2001; Myers 2018).

Many small rodent populations have erratic dynamics. However, voles and lemmings in the northern hemisphere, and particularly in Northern Europe, tend to have regular population fluctuations manifesting as cycles with a peak every 3–5 years (peak densities may attain 100–600 ind./ha, or 0.3–1.8 tons/km^2^). Elsewhere, small rodents can have larger outbreaks (1000–3000 ind./ha, or 1–5 tons/km^2^; Saunders 1986; Singleton et al. 2005, 2007; Leirs et al. 2010) with irregular intervals, usually, but not necessarily, exceeding 5 years (Singleton et al. 2007). Outbreaks occur both in the northern (Ostfeld et al. 1996; Jacob and Tkadlec 2010) and southern hemispheres, having major economic (Meerburg et al. 2009b; Singleton et al. 2010), conservation (Holland et al. 2015) and health impacts (Ostfeld et al. 1996; Meerburg et al. 2009a) both in developed and developing countries. In addition to the economic and health impacts of rodent outbreaks, population fluctuations in voles and lemmings are key for the functioning and structuring of boreal and arctic ecosystems (Ims and Fuglei 2005; Krebs 2011; Boonstra et al. 2016).

In this review, to improve our understanding of the mechanisms underlying the dynamics of populations, we compare small rodent cycles and outbreaks. There is a tradition in studies of population cycles to investigate the mechanisms driving the remarkably regular variation in density, which has resulted in a multitude of hypotheses explaining population dynamics (literature starting from Elton 1924, 1942, over Krebs 2013 and continuing). Ecologists studying outbreaks of small rodents have, however, often focused on the management of rodents due to their enormous impacts on humans through crop losses and disease transmission (Singleton et al. 2010).

The focus of the review is on population ecology. We have thus combined intellectual inputs from ecologists studying both population cycles and outbreaks in an attempt to achieve a synthesis. In our discussion, we highlight ten questions, the answers to which are essential for improving our perception of the various phases of the cycle or outbreaks. We do not provide a complete or specific list of open questions, but rather a selection of those major questions that require answers to better understand the generality of small rodent population dynamics.

## Outbreaks and cycles

### An overview

**Fig. 1:**
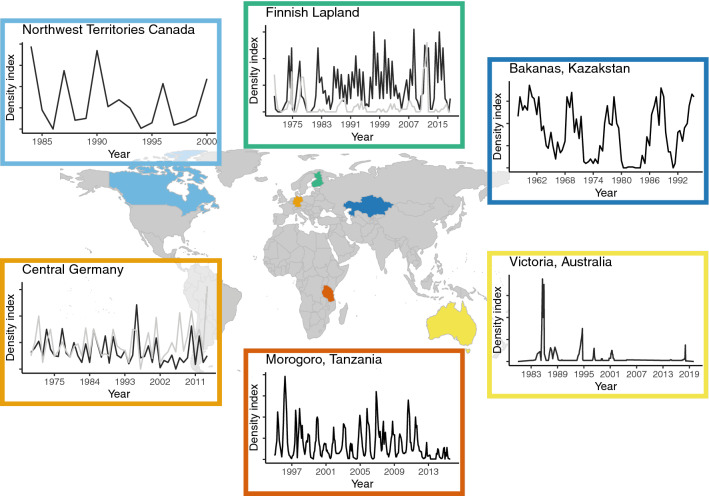
Small rodent population dynamics examples from representative long-term time series in different regions of the world: collared lemmings *Dicrostonyx torquatus* and brown lemmings *Lemmus trimucronatus* from northern Canada (top left; data sent by C.J. Krebs; Krebs 2011), two common vole species the bank vole (*Myodes glareolus*) = black line, and the field vole (*Microtus agrestis*) = grey line, from northern Finland (top centre) and Germany (below left), Great gerbils (*Rhombomys opimus*) in Kazakhstan (below right), Multi-mammate rat (*Mastomys natalensis*) in Tanzania, and house mouse (*Mus musculus*) in Australian grain-growing region. Discussion of the different population dynamics and references are found in the main text

Population cycles have been well described as periodic multiannual density fluctuations characterized by delayed density dependence in population growth rates (Stenseth 1999). The periodicity may be statistically derived from e.g. simple autocorrelations of abundance in time series data (Begon et al. 1996), autoregressive models (Stenseth 1999), or spectral and wavelet analyses (Elmhagen et al. 2011) and nonlinear time series analyses (Hsieh et al. 2008). In addition to periodicity, population cycles are often characterised by their astonishing amplitude, i.e. the difference between the maximum and minimum densities. During cycles, rodent densities typically increase by 2–3 orders of magnitude from the low phase, often with < 1 ind./ha, to the peak. Furthermore, the four phases of a population cycle, i.e. increase, peak, crash and low phase (e.g. Krebs and Myers 1974), are accompanied by various distinct phase-dependent features (Fig. 1).

Rodent outbreaks are less strictly analysed statistically as they occur largely at irregular intervals (Fig. 1). Nonetheless, their magnitude in both agricultural and forest landscapes can be so impressive that they have been described in the literature as early as the time of Aristotle (384–322 BC; Jacob and Tkadlec 2010). Rodent outbreaks have had dramatic economic, ecological, societal, and even political ramifications (Singleton et al. 2010). In recent times, rodent population outbreaks triggered by bamboo flowering and fruiting have been closely associated with changes in governments because of their devastating effects on vulnerable human communities of upland habitats in Asia (Aplin and Lalsiamliana 2010). Economically, global annual losses caused by rodents are consistently reported to be around 10–15% when pre-harvest (Meerburg et al. 2009b) and post-harvest losses (Belmain et al. 2015) are combined. Occasional outbreaks of rodent populations in developing countries have important implications for food and economic security from local to regional scales (Singleton et al. 2010). In developed countries, consequences are less drastic, but given that high rodent, density is often prevalent in specific regions and crops, the effect on businesses and supply chains can be dramatic (Jacob et al. 2014).

Defining a rodent outbreak is challenging because of the broad range of species and environments involved. Species that undergo outbreaks vary considerably in their population densities between non-outbreak and outbreak years. Long-term studies of house mice *Mus domesticus* indicate extremely low densities in most non-outbreak years (< 1 ind./ha), yet during outbreaks, population densities can be more than 3 orders of magnitude higher (Singleton et al. 2005). Saunders (1986) reported densities of > 3500 ind./ha and this is likely to be the norm for hundreds of thousands of square kilometres of wheat fields in Australia during a mouse plague. By contrast, other outbreaking species have moderate densities in non-outbreak years, while an outbreak entails increases in population densities of only 1–2 orders of magnitude. For example, African multi-mammate mice in Tanzania typically have seasonal peaks of about 150 ind./ha (Fig. 1), whereas in outbreak years densities can be tenfold (Leirs et al. 2010). A review of the bio-economics of five agricultural rodent pest species drawn from four continents highlights these differences in baseline densities and consequent outbreak trajectories (Stenseth et al. 2003).

Interestingly, the population dynamics of the same rodent species can have regular cyclic dynamics in some parts of their distribution, and irregular outbreak dynamics in other parts. This is true for the arctic lemming species, which appear to have a mix of cyclic and irregular outbreak dynamics within and between species (Ehrich et al. 2020) and over time (Henden et al. 2009). Field and bank voles (*Microtus agrestis and Myodes glareolus*) tend to exhibit population cycles in Fennoscandia (Hansson and Henttonen 1985), but less regular outbreaks in Central European deciduous forests. The exceptionally high population densities of especially forest-dwelling bank voles are related to bottom-up regulation by weather-driven beech mast (Imholt et al. 2015 Fig. 1). Common vole populations *Microtus arvalis* in Central Europe have also been shown to alternate between cyclic and non-cyclic dynamics, likely due to changes in habitat structure and land use (e.g. van Wijngaarden 1957). Both cycles and outbreaks in these *Myodes* and *Microtus* species are spatially synchronous across large regions and at least some features of their fluctuations are similar. According to Lambin et al. (2006), there may be no fundamental causal differences between cycles and outbreaks in Northern and Southern Europe.

### Phase dependent variation in population demography and behaviour

Phase dependent variation in the physiology and demography of cyclic vole and lemming populations has been well described. The most characteristic feature is the so-called Chitty effect, which involves vole body mass changes through a cycle with adults being 20–30% heavier in the peak phase than in the low-density phase (Chitty 1967; Boonstra 1994; Oli 1999, 2019; Sundell and Norrdahl 2002; Lambin et al. 2006). The smaller voles in the low phase tend to show delayed reproductive maturity. This phenomenon seems to be universal for cyclic vole populations. Animals are heaviest in the peak phase and produce the largest litters in the increase phase, while they are lightest and produce small litters in the decline and low phases. These demographic characteristics contribute to the asymmetric time series where both outbreaks and cycles show that the low phase may last up to several years, and the increased phase tends to be longer than the sudden crash and decline (Ginzburg and Inchausti 1997).

Chitty (1960) and later Boonstra (1994) proposed systematic changes in demographic population structure as the driving force of vole population cycles. Consequently, not only the quantity but also the quality of individuals may change during a cycle. Changes in the quality of individuals are likely to manifest as behavioural changes. Spacing is dramatically different at peak densities with more than 1000 ind./ha compared to the low phase with 1 ind./ha and less. During low density, it has been proposed that family groups in separated colonies may survive “by accident” and they would form the kernels to build up a local or area-wide increase again (Stenseth 1978; Glorvigen et al. 2013a, b). This well-documented phenomenon was even discussed as a possible driving force of population cycles (e.g. social fence hypothesis; Hestbeck 1982). The senescence hypothesis by Boonstra (1994) states that density-dependent social inhibition of breeding during the peak summer forces young to delay maturation until the next breeding season. Such density-dependent inhibition of maturation is quite common in territorial arvicoline rodents (e.g. Andreassen and Ims 2001).

Non-cyclic rodent outbreaks are predominantly driven by an elevation of reproductive rates some 6–9 months preceding a population outbreak. The conditions that trigger this atypical breeding pattern vary depending on the rodent species and the ecosystem. Nevertheless, species that have population outbreaks exceeding > 1000 ind./ha are typically characterised by an ability to extend their breeding season and/or to increase their production of young in response to climatic conditions and human agriculture that increase food supply. Such patterns have been reported in Australia (Singleton et al. 2001), Africa (Leirs et al. 1996), South America (Lima et al. 2003), Asia (Htwe and Singleton 2014), Europe (Jacob et al. 2014) and New Zealand (Ruscoe and Pech 2010) across many species. Apart from the breeding patterns, there are few generalities associated with the density-dependent and independent factors that influence the growth rates of species with erratic outbreaks (Stenseth et al. 2003).

### The seasonal structure of population dynamics

We refer to seasonality as the sequence of a breeding and a non-breeding season yearly. In high latitudes, seasons are defined by summer with vegetation growth and breeding of small rodents, and winter as a cold season with no, or only minor and exceptional, reproduction except for arctic lemmings where winter is the primary reproductive season. The length of the winter season varies with latitude and altitude with longer snow–covered periods polewards and upwards. Mediterranean climates in both the northern and southern hemispheres provide a comparable response, with usually more intense breeding of small rodents in spring and early summer, and low or absent breeding in the hot dry late summer and colder winter. In both cases, the non-breeding season is characterised by almost no photosynthesis, and thus practically no vegetation growth and no replenishment of food resources.

Stronger seasonality in high latitudes of the North shows that longer winters are associated with extended period lengths and larger amplitudes of the population cycles (Hansson and Henttonen 1985; Tkadlec and Stenseth 2001; Lambin et al. 2006; Taylor et al. 2013, but see Korpela et al. 2013). One piece of evidence for the importance of seasonality is the opposite geographical pattern in common vole fluctuations (Tkadlec and Stenseth 2001) as compared to the North–South gradient of Fennoscandian vole cycles (Hansson and Henttonen 1988). In northern Central Europe close to the Baltic Sea, common vole populations were more stable and increasingly cyclic towards southern Central Europe.

Also empirically-based modelling studies support the significance of seasonality as a determinant of the dynamics of cyclic populations (Bjørnstad et al. 1995; Stenseth et al. 2003; Kleiven et al. 2018). In arctic lemmings, the winters are key to reproduction while population densities often decline in summer (Ims and Fuglei 2005; Therrien et al. 2014). Due to the lack of reproduction during winter in voles, the strong, direct density dependence during winters necessarily involves winter survival. Seasonal and direct density-dependent mortality, together with direct and delayed density-dependent processes causing summer declines of populations, are necessary factors promoting multiannual cycles (Korpela et al. 2014). Examples are the population cycles of grey-sided voles *Myodes rufocanus* in Hokkaido, northern Japan (Batzli 1999; Stenseth et al. 2003), the cycle gradient of a whole vole community from northern to southern Fennoscandia (Hansson and Henttonen 1988; Hörnfeldt 2004), and cycles of the bank vole *Myodes glareolus* (Tkadlec and Zejda 1998) and the common vole *Microtus arvalis* in Central Europe (Tkadlec and Stenseth 2001; Pinot et al. 2016). The underlying process in this seasonal variation may be connected to predation or a limited amount of food produced during the preceding summer. Indeed, several studies confirm the significance of food resources for winter survival (Ylönen and Viitala 1991; Schweiger and Boutin 1995; Eccard and Ylönen 2001; Huitu et al. 2003, 2007; Boonstra and Krebs 2006; Johnsen et al. 2017; Soininen et al. 2018, but see Yoccoz et al. 2001).

Besides the significance of food resources, other resources related to the winter habitat may emerge as limiting factors. Larger territories will give access to a multitude of resources, such as food, nest sites, and mates. Korslund and Steen (2006) found that survival of tundra voles *Microtus oeconomus* increased with the increasing availability of the subnivean space. Similar results have been found for collared lemmings *Dicrostonyx kilangmiutak* and brown lemmings *Lemmus trimucronatus*. In arctic regions where snow is a strong limiting factor in the population growth of lemmings, amongst others the density of winter nests increased with snow depth (Reid and Krebs 1996; Reid et al. 2012; Bilodeau et al. 2013). Finally, Ylönen and Viitala (1985) found that bank voles aggregated in areas with brush vegetation before winter, which were also the areas with the thickest snow cover during winter. Winter aggregations benefit from a high level of social interactions (Ylönen and Viitala 1991), which promotes thermoregulation, i.e. heat and energy saving during mid–winter (Vickery and Millar 1984), and high reproduction at the onset of the breeding season in spring (Rémy et al. 2013; Andreassen et al. 2013; Radchuk et al. 2016). This may give rise to large growth rates in summer.

The picture of population fluctuations in non-seasonal environments in the tropics or in dry–temperate areas in the southern hemisphere is far fuzzier, as factors promoting resource availability and population growth are more stochastic (Leirs et al. 1997). Initiation of an outbreak seems to require the enhancement of food resources, which most often depends on e.g. rainfall and agricultural practices. In these environments, there are often distinct wet and dry seasons, which clearly determine the breeding seasons of rodents (Leirs et al. 1989; Massawe et al. 2011; Bâ et al. 2013). Unusually, wet periods or a prolonged rainy season result in longer or off-season breeding periods, with additional generations and therefore a multiplicative effect on abundance (Leirs et al. 1993).

However, extreme weather events with heavy rain and storms (Singleton et al. 2010) are not necessarily occurring regularly timed in the annual cycle. Thus, long-lasting droughts may maintain low population densities, while unpredictable rainfall periods boost irregular outbreaks of small mammals, like the house mice in dry-temperate Australia (Singleton et al. 2010).

This kind of irregularity is typical to tropical rats and other rodent outbreaks following bamboo masts in Southeast Asia, but nevertheless, outbreaks may also occur as not related to specific climatic events (Aplin and Lalsiamliana 2010; Belmain et al. 2010). Such climatic uncoupling has also been reported in beech mast-driven outbreaks of Central European rodent species (Reil et al. 2015). If extreme weather events like cyclones are followed by rapid plant growth in natural habitats and asynchronous, non-seasonal planting of rice in managed agricultural habitats, rodent densities and following agricultural damage may escalate rapidly. Outbreaks may also be favoured by the high mortality of predators due to cyclone hazards, leading to lower predation pressure (Singleton et al. 2010), but this idea has not been fully documented yet.

To conclude, seasonal effects may essentially shift rodent dynamics from an intrinsically stable regime with irregular fluctuations (generated by density-independent mechanisms) to larger–amplitude and periodic cycles influenced by density-dependent mechanisms (Stenseth et al. 2003). Irregular outbreaks, on the other hand, seem to be primarily linked to stochastic weather events.

## Phase dependent effects and related questions

We acknowledge a recent statement by Oli (2019) that “Solving the enigma of population cycles may necessitate identifying factors and processes that cause phase-dependent demographic changes and performing conclusive experiments to ascertain the mechanisms that generate multiannual density fluctuations”. Hence, in the following we discuss the mechanisms shaping population dynamics of voles and lemmings, for which four cycle phases, i.e. increase, peak, crash and low phase, can typically be identified. We will, however, also consider population outbreaks whenever this is feasible, and comparison may provide relevant insight.

### The increase phase

The literature regarding small rodent population cycles mostly focuses on the crash phase and the ensuing low phase, and on the factors that may cause these (e.g. Boonstra et al. 1998). Surprisingly, much less effort has been devoted to studying processes of populations escaping regulation from low densities and transitioning into extended periods of increasing density (Hein and Jacob 2015).

The transition of a stable, low-density population into one with density independent population growth is facilitated by a shift in population demography, such that reproductive rates and/or immigration become greater than mortality and/or emigration. Increasing population densities of small rodents in favourable environmental conditions and low intraspecific competition can be easily explained by the intrinsically high rates of sexual maturation and reproduction (Turchin and Ostfeld 1997). The challenge is, however, to identify the factors that define good environmental conditions which allow the increase. This is particularly relevant, as both the rates of increase and the duration of the increase phase vary substantially from one peak to the next, suggesting that also environmental conditions vary (see e.g. Boonstra et al. 1998).

Firstly, adequate food resources are a necessity for population growth. Food resource availability is, by and large, governed by abiotic conditions. In low and early increase phases, densities are often very low. Therefore, competition for high-quality food is likely to be negligible. At high latitudes, reproduction in cyclic small rodent populations commences at the onset of plant growing season in spring after several months of winter (e.g. Prévot-Julliard et al. 1999), except for the arctic lemmings mostly breeding under sub-niveal protection (e.g. Ims and Fuglei 2005). At lower latitudes, rainfall determines the condition of vegetation, and hence acts as a pivotal limiting factor for small rodent population growth. This is especially true for arid regions (see Bennison et al. 2018) and for semi-arid regions with seasonal rainfall (Tann et al. 1991; Leirs et al. 1994; Luque-Larena et al. 2013). In desert environments, patterns of precipitation are often highly unpredictable, and often affected by large–scale climatic anomalies such as the El Niño Southern Oscillation (Lima et al. 1999). Small rodent reproduction can also be strongly impacted by pulsed variation in food availability (so-called mass occurrences) in more productive areas, such as in the case of European beech *Fagus sylvatica* (Jensen 1982; Wolff 1996) or several bamboo species (Belmain et al. 2010; Htwe et al. 2010).

Secondly, small rodent population growth cannot be achieved in environments in which the mortality effects of predation override rates of reproduction. According to the specialist predator hypothesis (Andersson and Erlinge 1977), cyclic vole populations can sustain many predators during the peak and crash phases. However, after vole densities remain low for a sufficient time, predator numbers dwindle due to either starvation or emigration (Norrdahl and Korpimäki 2002), providing small rodents with enemy–free conditions in which to procreate. Such settings are typical for Northern Europe.

In temperate areas vertebrate communities are more complex, containing more of both alternative prey species and generalist predators that prey on them. The latter has been shown to have a stabilizing effect on vole population dynamics (Hansson and Henttonen 1985; Hanski et al. 1991), partly by a considerable shortening of the time window with enemy-free conditions during which rodent population growth is expected to take off. In small mammals exhibiting irregular population outbreaks in arid regions, the periods between peaks are often too long for predator populations to subsist in moderate densities (Sinclair et al. 1990), thus restricting their impact to the proximity of the peak itself (Meserve et al. 2003). However, not all species behave in the same way: Lima et al. (2003) showed that in the leaf-eared mouse (*Phyllotis darwini*) in Chile, population growth rate throughout the year is dependent on survival (for which predation is thought to be important), while for the multi-mammate mouse (*Mastomys natalensis*) in Tanzania, changes in reproductive output are much more important for population growth.

Thirdly, the intrinsic behavioural and social processes operating within populations of both cyclic and eruptive species vary considerably during different phases of their dynamics. Several of these processes may be beneficial during increasing population densities. For example, the increase phase is initiated by the demes of animals distributed in high-quality patches of the landscape (Sundell et al. 2012). Resource patchiness may promote social behaviour in females and enhance their reproductive success compared to solitary territorial females (Ylönen et al. 1988; Ylönen and Viitala 1991; Lambin and Yoccoz 1998; Sutherland et al. 2005; Rémy 2011). The benefits may manifest through communal breeding and thermoregulation, particularly during winter (Hayes 2000; Gilbert et al. 2010), and shared protection against infanticide (Wolff 1993; Ylönen et al. 1997). The early phases of the increase will be associated with dispersal and rapid colonisation of vacant habitat patches (Glorvigen et al. 2013a, b), as dispersal is inversely density-dependent in voles (Andreassen and Ims 2001). The correlation between amicable social behaviour and population growth rates have been described for several species of rodents, such as house mouse (*Mus* spp.; Krebs et al. 1995; Sutherland et al. 2005), yellow-necked field mouse (*Apodemus flavicollis*; Bogdziewicz et al. 2016), and *Myodes* and *Microtus* voles (Ylönen et al. 1990; Andreassen et al. 2013 and references therein). These species inhabit various biomes in the world and vary in population dynamics from occasional outbreaks to population cycles.

In conclusion, it seems to be obvious that small rodent population increases are associated with abundant food resources, enemy-free conditions, and certain types of social behaviour. However, there are details regarding the increase phase that is currently poorly understood, which can be broadly summarized into the essential question related to the increase phase:

1. *What factors determine the rate and the timing at which rodent populations increase, and what defines the length of the increase phase?*

The rate of population growth, assuming closed populations and minimal mortality, is a function of reproductive output. Small herbivorous mammals subsist primarily on a relatively poor quality diet, particularly regarding the intake of nitrogenous compounds, and especially essential amino acids (Mattson 1980) that are crucial for maintenance and reproduction. Certain amino acids are a limiting factor for per capita reproductive output in cotton rats *Sigmodon hispidus* (Webb et al. 2005). This indicates that diet quality may well affect population growth rates of both cyclic and eruptive species during the increase phase. However, this association has received virtually no research attention, let alone how diet quality varies in response to the weather. In arid areas, the quantity of food clearly influences the population growth rates of small mammals. Such an association may also affect cyclic small mammal populations in more predictable growing regimes, e.g. in Northern Europe, where dry and hot summers often appear to inhibit vole population growth.

The quantity and quality of food resources are likely to have major effects also on the duration of population increase. In general, multivoltine small mammals with several litters in one—and often the only—breeding season of their lifetime, require long growing seasons or need to breed in several consecutive summers (Prévot-Julliard et al. 1999), to reach the absolute carrying capacity of the population. As a seasonal effect, it is obvious that environments that exhibit long winters also have a short growing season.

Furthermore, rodents depend heavily on intestinal microbes for the digestion of their bulky and cellulose-rich food (Ley et al. 2008). The composition of the rodent intestinal microbiota is greatly affected not only by their diet (Kohl et al. 2014), but also by pathogens and parasites (Guarner and Malagelada 2003), and this, in turn, may reflect upon the immune system (Guarner and Malagelada 2003). The role of such changes in the intestinal microbiota on phase-related changes in rodent demography has, to our knowledge, never been investigated until the work of Li et al. (2019).

### The peak phase

Population peaks largely determine the attained density amplitude of the population. They are reached when mortality first equals then exceeds reproduction, to prevent a further increase in density. Immigration and emigration are supposed to be in balance during the peak phase, which is a reasonable assumption as small rodent fluctuations are commonly spatially synchronous over vast areas (e.g. Sundell et al. 2004).

**Fig. 2:**
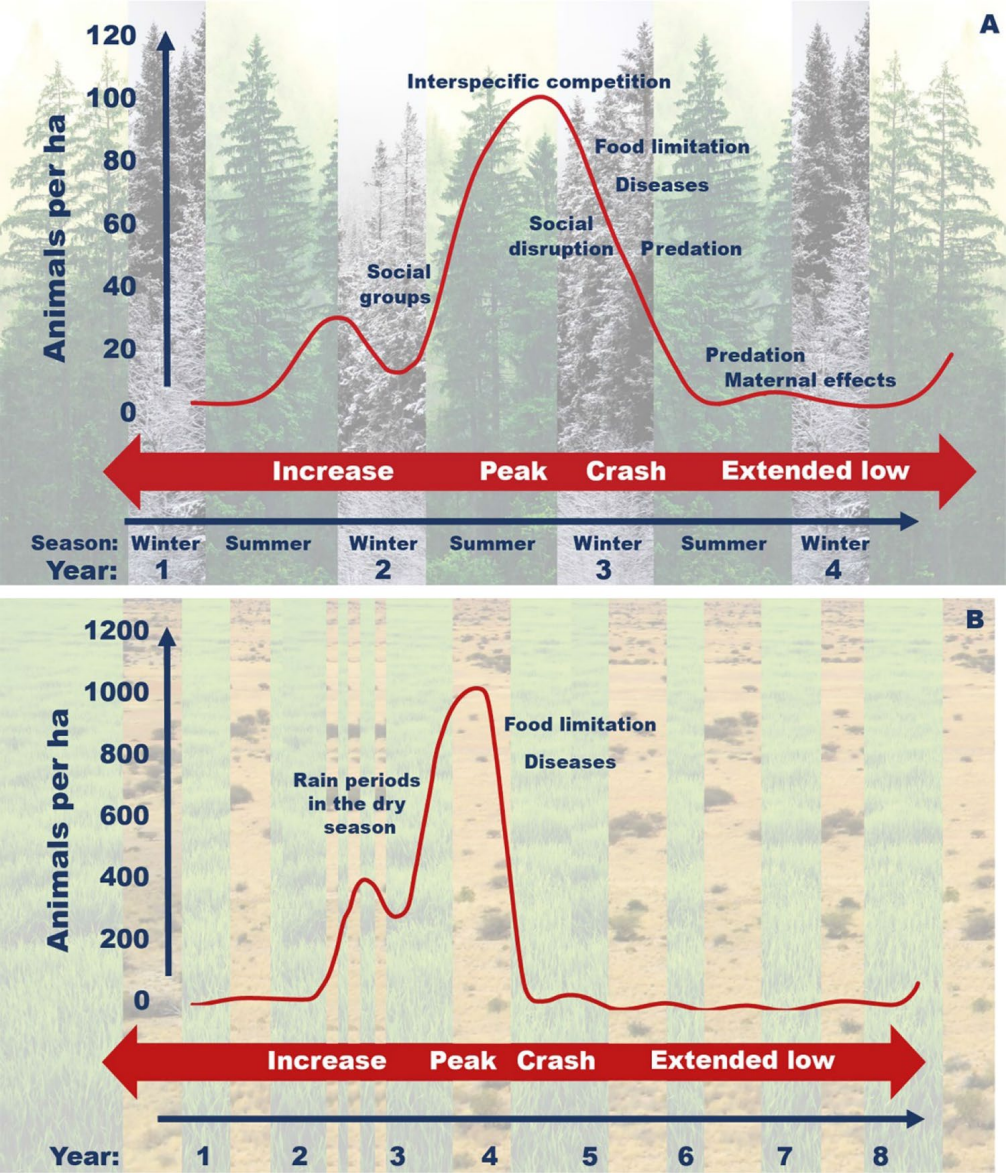
Characteristics of vole population cycles in the northern hemisphere (**a**) and mice outbreaks in the southern hemisphere (**b**). Seasonality connected to reproductive and non-reproductive periods. Winter is the non-reproductive season in the northern hemisphere and the dry season in the south

Peak densities typically vary substantially from one cyclic peak or outbreak to the next, also for the same population in the same area (Fig. 1). Variation in the limiting factors is associated with changes in the carrying capacity of the environment, as determined by either abiotic conditions (e.g. weather or habitat availability), or biotic factors such as food resources or predation. Variation in weather may affect primary production and biomass accumulation, which in turn affects the amount of available food resources or the extent of foraging and breeding habitats. For example, a warm and dry summer may greatly limit the growth of rodent food plants, resulting in a low amount of accumulated food resources with which to overwinter (Korpela et al. 2013) (Fig. 2).

Most of the factors proposed to cause cyclic dynamics in small rodents can also influence peak density levels and even cause cessation of population growth. These include competition, predation, reduced food availability and quality, pathogens and parasites, stress, and quality of individuals, as well as social factors such as infanticide (e.g. Stenseth and Ims 1993; Oli 2019). These factors potentially limit population growth, but they do not necessarily regulate populations, i.e. they do not cause the cyclic dynamics per se. This problem may be exemplified by the multiannual fluctuations of northern voles, which are thought to be mainly caused by delayed density-dependent factors such as predation by specialist predators (e.g. Korpimäki and Norrdahi 1998; Hanski et al. 2001; Korpimäki et al. 2002). However, even the classic predator–prey models inherently require some direct density-dependent process to slow the prey’s population growth, so that predators with their much lower reproductive potential can “catch” the prey population and cause the subsequent crash (e.g. Hanski et al. 2001). Yet, it is important to note that, in seasonal environments, the predator functional response alone can generate direct dependence even when predator species express various functional responses (e.g. Gilg et al. 2003). Huitu et al. (2003) identified winter food resources as such as a direct density-dependent limiting factor in a two-factor experiment manipulating both predation and winter food supply. The great gerbil *Rhombomys opimus* in the Central-Asian steppe in Kazakhstan exhibits cyclic population fluctuations that are linked with the flea burden on these rodents and epizootics of *Yersinia pestis* plague (Reijniers et al. 2014). Meanwhile, Kausrud et al. (2007) showed that climate forcing synchronizes the dynamics of these gerbils over large geographical areas. In ecological population models, many of these factors can co-occur, and their relative strength is almost impossible to gauge or even parameterise. Hence, this “untouchable clump of factors” is often incorporated as a black-box in the models (Stenseth 1999).

There are many additional direct density-dependent factors that may contribute to population fluctuation patterns. These may be related to predation, for example, selective predation on the reproductive part of the prey population (Cushing 1985), changes in the predator spectrum due to shifts in prey activity patterns (Halle and Lehmann 1987; Halle 1993), indirect predation effects (Ylönen 1994; Ylönen and Ronkainen 1994; Sheriff et al. 2009), fast functional response of the generalist predators (Hanski et al. 1991), and fast numerical response of nomadic avian predators (Sundell et al. 2004). Little is known about the many potential interactions of the multiple factors, as this kind of network is hard to control in experimental studies. Food and predation/parasite—interactions are the most studied of such interactive effects (e.g. Pedersen and Greives 2008; Haapakoski et al. 2012; Forbes et al. 2015), but other or multifactorial interactions are hardly touched.

The shape and magnitude of the peak phase of population cycles vary considerably between species (Turchin et al. 2000; Turchin and Batzli 2001), for example between the sympatric northern species *Myodes rufocanus* and *Lemmus lemmus* (Ims et al. 2011)*. Myodes* populations, as many vole species in general, have cycles with blunt, often two-year peaks (Ylönen 1988) compared to *Lemmus* populations that have more angular, saw-toothed cycles with higher maximum densities (Turchin et al. 2000; Ehrich et al. 2020). These differences are suggested to be due either to different causal trophic interactions (predator–prey in *Myodes* and plant–herbivore in *Lemmus*; Turchin et al. 2000), or to winter breeding (most prevalent in *Lemmus*; Ims et al. 2011). Andreassen et al. (2013) suggested that different social organisations between the species or genera might be linked to the shape of cycles, with sharp, high–amplitude cycles being typical for species with male territoriality and female sociality. Thus, *Microtus* species tend to have sharper cycles than *Myodes* species, where the social system is characterised by female territoriality (Kalela 1957; Viitala 1977; Ylönen 1988). The social system of lemmings is more flexible and may rather depend on territorial males (Heske and Jensen 1993). Moreover, the extreme shifts in dispersal and social behaviour observed in *Lemmus* (specifically in *L. lemmus*; Stenseth and Ims 1993) fit well to the suggestion that the shape of the cycle may be linked to behaviour and social organisation.

The absolute height of the peak in animal numbers is determined to a large extent by food resources. As an example, the numbers of house mice in Australia, breeding in grain fields, reach even thousand(s) of individuals per hectare during outbreaks (Singleton et al. 2005). In Europe, the herbivorous *Microtus* voles inhabiting agricultural landscapes reach two- to three-fold higher densities than the granivorous *Myodes* voles (Henttonen 2000). Exceptionally good food supply may promote disruption of the social system and territorial behaviour, which normally controls the numbers of breeding females as observed by Ylönen et al. (1988).

To compile this section, a rather simple essential question arises in connection with the peak phase:

2. *What are the factors that determine the height of the density peak, and how do they interact?*

A thorough understanding of the continuous variation in density amplitude for each cyclic or eruptive population in any geographical region and habitat productivity could bring new insights into population dynamics. More specifically, it is important to recognize those direct density-dependent factors that hinder population growth near the peak densities. An additional question is whether the social structure of peak density vole populations remains the same as in increasing populations, or whether some kind of change or disruption of the social structure occurs, which would enable more females to breed in a stressing high–density environment. This kind of loss of social control in breeding during very high densities was observed by Eccard et al. (2011).

### The crash phase

The decline of the population after a peak or outbreak is often abrupt and dramatic, and therefore it is called a crash. The crash phase has received the most attention in the literature and it is indeed critical for the understanding of small rodent population dynamics (Tkadlec and Zejda 1998). In cyclic small rodent populations, the crash often starts in late summer or fall and extends into winter and the following breeding season (Krebs and Myers 1974; Hansson and Henttonen 1988; Huitu et al. 2003; Pinot et al. 2016; Johnsen et al. 2017). In many cyclic populations, the initial autumn/winter crash is followed by summer declines strongly affected by specialist predation (Henttonen et al. 1987; Hanski et al. 1991). However, summer declines during the population crash are also observed in cyclic populations of the field vole in Kielder Forest in UK (Lambin et al. 2000), where virtually no strictly specialist predators are present, and in other non–cyclic rodent populations in Central Europe (Giraudoux et al. 2019).

The crash itself also has most often been connected to specialist predators, especially to small mustelids that can enter the holes and cavities of small mammals, their nests and the subnivean space in winter (Norrdahl and Korpimäki 1995; Boonstra et al. 2016; Ylönen et al. 2019). The predator hypothesis is supported by mathematical models (e.g. Hanski et al. 2001) as well as by experimental studies (Korpimäki 1993; Klemola et al. 1997; Korpimäki and Norrdahi 1998). Although no one denies that (specialist) predators contribute greatly to the crash of small rodent populations, some authors have combined predation, or other extrinsic factors, with intrinsic factors as potential enforcers of the decline. For instance, Andreassen et al. (2013) suggest that predation disrupts the social system, followed by intraspecifically induced mortality such as infanticide (see e.g. Ylönen et al. 1997; Andreassen and Gundersen 2006; Opperbeck et al. 2012). The problem with this framework is, however, that it considers a typical scenario for a crash during the breeding season with strong social interactions between territorially breeding animals (e.g. Ylönen et al. 1990). Nevertheless, in most crashes, the steepest decline in numbers is observed during the winter when the territorial behaviour of voles is expected to be relaxed and animals rather aggregate for thermoregulation (e.g. Ylönen and Viitala 1985, 1991; Sipari et al. 2016). Whether the mortality rate throughout winter is constant is yet to be assessed since most studies do not measure population changes throughout the winter but compare before and after winter numbers.

Intraspecific competition during winter for food resources has repeatedly been suggested as a factor limiting the growth of vole populations at peak phases. Obviously, as food resources are not being renewed during the winter, food depletion and deterioration of its quality can easily be regarded as a contributing factor also to the crash phase, as suggested by Boonstra and Krebs (2006) for red-backed voles *Myodes rutilus*. Several experimental studies also show that supplemental feeding during winter can create high autumn densities in local patches in red-backed voles (Schweiger and Boutin 1995), advance breeding of bank voles *Myodes glareolus* in spring (Eccard and Ylönen 2001; Ylönen and Eccard 2004), and reduce territorial behaviour in bank voles (Ylönen and Viitala 1991). It may even prevent winter crashes of bank voles (Johnsen et al. 2017) and, when supplementary feeding was combined with the elimination of predation, in the field vole *Microtus agrestis* (Huitu et al. 2003).

In support of winter food limitation, Huitu et al. (2007) found evidence for deterioration in the physiological condition of field voles in the winter of the decline phase compared to the winter of the increase phase. The poor condition of these voles may expose them to diseases, parasites and/or predators, leading to a feedback loop of increasing mortality (Beldomenico et al. 2008). Intraspecific competition for food resources may also explain why larger activity ranges are beneficial for survival during winter (Johnsen et al. 2018).

Related to this is the effect of the larger animals in peak years as described by the Chitty effect (see above; Chitty 1967). It has been shown that small rodents have a physiological optimal winter body mass that is species-specific (Iverson and Turner 1974; Wiger 1979; Aars and Ims 2002). The characteristic of larger animals during the peak may be age-related, as younger cohorts have been inhibited from maturation to the adult subdivision of the population (Andreassen and Ims 2001). The large peak animals may struggle to survive the winter because they are physiologically “too big” and energetically sub-optimal for the limited food resources. This may, together with social intolerance in males, be one reason why the survival of males is generally lower over winter as compared to females (e.g. Klemme et al. 2008; Haapakoski et al. 2012; Sipari et al. 2016).

Depletion of food resources or some specific food items needed in only small amounts (Aulak 1973; Andreassen and Bondrup-Nielsen 1991) may also explain the continuing decline into the following summer, although this has been refuted experimentally by Klemola et al. (2000b). Furthermore, the challenge with the food hypothesis is, however, to understand how this can affect the whole small rodent community consisting of species with markedly different diet requirements, like seeds in *Myodes*, graminoids in *Microtus* and mosses in *Lemmus* (Hansson and Henttonen 1985); but see (Soininen et al. 2017b).

Limited food resources may also interact with predation and/or pathogens and diseases to further reduce population numbers (Huitu et al. 2003). Studies focusing on the mortality causes in cyclic vole populations support the strong effect of predation, as Steen (1995) observed in cyclic tundra voles *Microtus oeconomus*, and Norrdahl and Korpimäki (1998) for radio–collared *Microtus* voles. In studies where predation rates were precisely estimated, the population growth of arctic lemmings in summer was limited by predation pressure, e.g. by predatory birds (Therrien et al. 2014). Nevertheless, during the crash phase, predators are likely to act compensatively, i.e., kill starving or diseased individuals that would die anyway. Relatively few animals are found dead during a crash except for Norwegian lemming *Lemmus lemmus* where surplus killing can translate into many carcasses (Steen et al. 1997); for voles, however, with crashes mostly occurring during winter, scavenging by many predators can be a simple reason behind the absence of dead animals found in spring. So, the essential question related to the crash phase can be framed as:

3. *How does the population demographic structure affect the crash phase?*

The population crashes of eruptive species in the southern hemisphere are more rapid and impressive than the decline in vole and lemming populations. House mouse populations literally crash synchronously within weeks over thousands of square kilometres (occasionally as fast as 90% reduction of animals within a week; Singleton et al. 2007), including around grain stores where there is still ample food.

Changes in spacing behaviour of house mice during the development of high population densities and during the rapid population crash in wheat fields in Australia indicate that they are highly territorial during the breeding season of an increase phase. These changes in spacing behaviour also signal that there is a complete breakdown of social and anti-predatory control mechanisms once populations are high and during the rapid decline in population numbers (Chambers et al. 2000; Ylönen et al. 2002; Jacob et al. 2004; Sutherland and Singleton 2006). This resembles the breakdown of social breeding control in the bank vole during high densities of mature females (Ylönen 1988; Eccard et al. 2011) and may indicate that population growth to very high densities is a combined effect of resource availability and changes in population social structure. Following a crash, eruptive species like house mice may be under pressure by a combination of caloric and disease stress (Singleton et al. 2007), and finally doomed by predation on sick and weak individuals.

Although the scientific literature and experimentation regarding both cyclic and eruptive small rodent populations have focused on the crash phase, we have still not reached a consensus on conclusive explanatory factors. The Chitty effect characterising individuals in peak phases has received little attention for decades, and we know even less about changes in the population demographic structures in the more eruptive tropical populations. In the latter populations, however, the increase is often very fast as well, in immediate response to stochastic climatic events, and both increase and crash often happen within the same year, not allowing for a shift in demography. Population demography and the structure in the population (e.g. sex ratio, age structure and body mass) shift through the population cycles. It is about time to solve eventual mechanisms for the effect of population demography structure during the crash phase, for instance through physiological constraints in body mass, or senescence (Boonstra 1994).

### The low phase

A feature as remarkable as the density peaks in the cyclic population is that densities do not start to grow immediately after a crash despite ample food resources and low intraspecific competition. This so-called extended low phase has initiated much research on population dynamics. For populations to stay stable at low densities over a longer period, mortality and reproduction, as well as immigration and emigration, should be in balance. Hence, since immigration and emigration are primarily considered to compensate for local density disparities (see above), there has to be a factor that lowers reproduction and/or increases mortality, preventing the population from increasing.

In cyclic populations, the low phase may last 2–3 years before the populations start to increase again (Boonstra et al. 1998). The same phenomenon is observed in eruptive populations that have unpredictably long low phases (most often 5–10 years; Singleton et al. 2007). We need, however, to distinguish between rodent outbreaks in arid areas like Australia vs. temperate Europe where low phases may also be long, but not necessarily so (Jacob and Tkadlec 2010). Multi-mammate mice revert to “normal” seasonal fluctuations after the end of an outbreak and that condition may then last for up to several years until a new outbreak is triggered (Leirs et al. 1996).

Food availability was rejected as an explanation for the extended low phase by Boonstra et al. (1998); and experiments have not found delayed effects of food availability on population growth (Turchin and Batzli 2001). Food becomes a limiting factor only at higher densities (Huitu et al. 2003), and previous overgrazing does not prevent vole populations from increasing (Klemola et al. 2000b). Several food plants of cyclic voles induce phytochemical defences in response to intensive vole grazing (Massey and Hartley 2006; Reynolds et al. 2012; Huitu et al. 2014), and some of these responses are delayed. However, no evidence exists for any universal induced defence substance, applicable across cyclic small rodent taxa (Soininen et al. 2017a).

The lack of universality also applies to rodent pathogens. Although a growing number of studies are reporting significant negative effects of pathogens on the survival of its host (e.g. Soveri et al. 2000; Kallio et al. 2007; Burthe et al. 2008), no pathogen can be common and widespread enough to be responsible for the delay in host population growth at low densities. Pathogen prevalence is generally highest when their hosts reach large densities (e.g. Singleton et al. 1993, 2000), but whether density alone or a combined effect with the cycle phase drives pathogen prevalence remains unsolved. A recent study using cyclic populations by Forbes et al. (2014) identified delayed density-dependent patterns of orthopoxvirus (likely cowpox) prevalence in field voles in Finland, implying that this pathogen may contribute to the low phase of the cycle.

For cyclic populations, Boonstra et al. (1998) concluded that predation and maternal effects are the most likely explanations for the extended low phase. A delayed numerical response of the predators continues to inflict mortality on the population in the low-density phase. A density increase of the prey is impossible until the predation pressure subsides (Henttonen 1985; Korpimäki 1986; Sonerud 1988), which happens when predator numbers are reduced due to mortality, cessation of reproduction and emigration, or if the predators switch to other prey species.

Reduction or removal of predators in the low phase should shorten its duration. Predator removal experiments have been conducted, but they have seldom covered the period between decline and increase of prey populations. One comprehensive experiment exists (Korpimäki et al. 2002) which showed clear effects of predator removal on the abundance of voles in all studied cycle phases, albeit without a marked impact on the length of the low phase. The reason for this might have been that the reduction of all vole predators was conducted only during the breeding season. In two studies (Klemola et al. 2000a; Huitu et al. 2003), all vole predators were excluded from fenced areas during the low phase, with a similar result—fenced populations increased while unfenced control populations remained at a low level.

Several attempts have been made to employ predators as biocontrol agents, e.g. to prevent crop damage associated with outbreaks (e.g. Mahlaba et al. 2017). These attempts often generate high predator densities, but generally fail to keep rodent numbers down. However, most of these rodent populations were not cyclic. Apart from Duckett (1991) and Kay et al. (1994), there is no convincing empirical field data to suggest that promoting the presence of avian predators (by nest boxes and perches) leads to lower rodent abundance or reduced damage to crops (Labuschagne et al. 2016).

Predation may also have indirect, delayed effects on voles through maternal effects, involving e.g. stress. This is likely to affect in particular individuals of the low phase of cycles, as has recently been shown for snowshoe hares *Lepus americanus* (Sheriff et al. 2009; Krebs et al. 2018). Furthermore, it is important to note that some 10 years ago classical Mendelian heritability of individual traits was assumed a prerequisite for intrinsic effects to be relevant for population regulation. The recent advent of epigenetics has dramatically changed this view (e.g. Bossdorf et al. 2008).

We largely share the views presented by Boonstra et al. (1998) over two decades ago, and conclude that the extended low phase of cyclic small mammal populations is indeed most likely caused partly by extrinsic predation, but partly also by delayed intrinsic, inter-generational effects of predation pressure that modify the quality of individuals living at low densities. Future studies should aim to determine the relative importance of delayed effects of predation, and other density-induced stressors such as social and nutritional stress, through direct and indirect pathways on the demography of small rodent populations. As the evidence on intergenerational effects of early-life environment on survival and reproductive success is accumulating also from voles (Bian et al. 2015, van Cann et al. 2019a, b), more emphasis should be placed on quantifying the phenotypic and (epi-)genetic characteristics of individuals in different phases of the population cycle.

Hence, an essential question for future research on the low phase is:

4. *Why do populations not begin to grow immediately after a crash, and are pathogens or maternal effects relevant ingredients for the extended low phase of cyclic populations?*

For populations with eruptive dynamics, the periods between outbreaks likely represent a normal, more or less stable state of populations, in which densities are not particularly low, compared to most cyclic populations in the North.

## Small rodent populations in a changing world

The world is changing rapidly, also for small rodent populations. Besides the obvious changes of global warming and the increased occurrence of extreme weather events, land-use change is another important factor that could influence the dynamics, health and resilience of small mammal populations. Below, we discuss some related aspects that potentially are important future research issues for ecologists of small rodent population dynamics.

### Climate change

In eruptive populations of the southern hemisphere and temperate Europe, rodent population increases to abnormally high densities are often associated with stochastic climate events or irregular resource changes. In Tanzania, unusually abundant rainfall early in the wet season triggers early reproductive maturation in multi-mammate mice *Mastomys natalensis*. This produces an additional generation within a year, resulting in a tenfold production of young and outbreak densities (Leirs et al. 1993). In Southeast Asia from 1996 to 1999, unusual rainfall patterns led to asynchronous planting of rice crops, followed by rodent population outbreaks each year (Huan et al. 2010). Similarly, the high degree of asynchronous planting of new rice crops over a large area in Myanmar after the cyclone Nargis in 2008 has been suggested to be the most likely contributing factor to the massive population outbreak of *Bandicota* species some 15–18 months later, in areas where outbreaks had never been experienced before (Htwe et al. 2013).

In Central Europe, beech mast triggers bank vole population outbreaks in the following year (Tersago et al. 2009; Reil et al. 2015). The weather conditions favourable for beech mast are likely to occur at higher frequencies in the future due to climate warming. In New Zealand, mouse populations erupt during a beech mast and it has been reported that the magnitude of change in mean summer temperature between consecutive years can predict mast events. Therefore, the frequency of outbreaks of mouse populations in New Zealand forests, and perhaps also of bank voles in beech forests in Europe, may rise with increased variance predicted in climatic events (Imholt et al. 2015; Holland et al. 2015).

There are two major scenarios of how small mammals in the North are affected by climate change. Enhancement in habitat productivity due to warming and increased precipitation may result in agricultural intensification and related land-use changes (cf. Cornulier et al. 2013; see below). On the other hand, winters are predicted to become more unstable and the duration of permanent snow cover shorter, which affects the life of ground-dwelling small mammals and food webs in many ways (Penczykowski et al. 2017). Currently, approximately one-third of the world’s land surface is covered by snow during winter (Lemke et al. 2007). Snow cover provides thermoregulatory advantages in the insulated subnivean space, shelter for nest sites, and physical and visual refuge from predators. Hence, shorter periods with snow cover are most likely to affect winter survival of voles and lemmings negatively.

Predation by specialist predators, especially the least weasel *Mustela nivalis* and the stoat *Mustela erminea*, is suggested to be a key factor promoting the population crash and causing extended low phases (see above). Both species belong to the group of vertebrates in the North changing from dark or brownish summer pelage to a white winter coat. Late and unpredictable onset of snow cover and its earlier melting could increase the vulnerability of individuals with a mismatched white coat colour due to intra–guild predation by larger mammalian predators and resident owls. This, in turn, may have dramatic effects on vole dynamics (Ylönen et al. 2019) and further cascading trophic effects at the ecosystem level (Terraube et al. 2015).

Empirical studies on the interaction between climate and predation are scarce. There are two northern-boreal examples of severe changes in vole dynamics, the temporal disappearance and return of vole cycles together with weasel disappearance in Finnish Lapland (Fig. 1, Henttonen et al. 1987, Magnusson et al. 2015), and the low densities of grey-sided voles *Myodes rufocanus* and field voles *M. agrestis* in Sweden (Hörnfeldt 2004; Hörnfeldt et al. 2005). Dampening of the Swedish grey-sided vole cycle is more clearly attributed to changes in forest landscape structure (Hörnfeldt 2004; Ecke et al. 2006; Magnusson et al. 2013, 2015), while dampening of the cycles of the field vole along with their recent recovery, are more likely related to a climatic driver (Magnusson et al. 2015). In contrast, the disappearance and subsequent return of vole cycles in Finnish Lapland seem to be due to a more complex network of changing seasonality and predator–prey interactions in a whole rodent community (Henttonen 2000; Ylönen et al. 2019). Several arctic lemming populations showed perhaps the most compelling examples of collapsing cycles in recent years (Ims et al. 2008), e.g. in North-Eastern Greenland (Schmidt et al. 2012). It is possible that several observed collapses are actually transitions to non-stationary population dynamics as detected by analysing hundred-year long time series (e.g. Henden et al. 2009). Such transitions between stationary and non-stationary can be triggered by several factors, such as non-linear trophic dynamics (Hastings et al. 2018; Clark and Luis 2020; Blasius et al. 2020).

The examples above show how global warming and more variation in extreme weather may change the dynamics of small rodent populations. In the northern hemisphere, a warmer climate may improve habitat quality, while a drier, and more unfavourable climate is expected in the southern hemisphere. How this will affect population dynamics is not obvious. One possibility is that with a warming climate, northern populations would begin to exhibit similar types of erratic outbreak dynamics as currently observed in southern populations. For the South, we already know that rainfall is one of the most important determinants of outbreaks today because it increases primary productivity and food availability, as exemplified for instance by the *Mastomys* rats in Africa (Leirs et al. 1996). As a response to a drier climate, outbreaks may occur more rarely in the southern hemisphere. Yet, those outbreaks may be more dramatic than before, since a long dry spell of several years, broken by an unexpected wet period, may result in uncontrolled growth of the rodent population; all supported by abundant vegetation growth due to the build-up of a rich seed bank and soil nutrients and the absence of predators as suggested by Fiedler (1988).

The above assumptions are reasonable but speculative, since they are projections into possible future complex developments while evidence is missing up to now. An essential question related to global climate changes to follow up is, therefore:

5. *How will climate and land-use change affect small rodent dynamics in both cyclic and eruptive populations?*

The most regularly cyclic populations are found in the northern hemisphere, while outbreaks are more typical from Central Europe to the tropics and Australia. However, there are examples from temperate or arid Europe on eruptive types of fluctuations, resembling a hybrid between cycles and outbreaks (Luque-Larena et al. 2013; Reil et al. 2015). Whether this is a result of cyclic population dynamics eventually turning into more eruptive dynamics, possibly induced by climate change, is plausible but remains to be verified. However, not all rodent species respond in the same way or at a similar pace to climate change (e.g. Gilg et al. 2009). Furthermore, changes in temperature affect other climatic components such as precipitation differently in different parts of the globe, which affects the intervals and intensity of outbreaks. It is therefore too early to cast global predictions regarding the potential effects of climate change on small rodent dynamics.

Of note, climate change is also a major driver of changes in rodent-borne disease patterns (Kausrud et al. 2010; Voutilainen et al. 2012; Altizer et al. 2013; Khalil et al. 2014). Increased trafficking and human encroachment into wildlife habitats will probably accelerate the spread of parasites around the world, also increasing the incidence of rodent-borne zoonotic outbreaks.

Land-use change may influence the presence and absence of small mammal species, or influence their temporal and spatial dynamics directly or indirectly. Populations of *Microtus agrestis* in the UK, in Sweden and in Finland are cyclic. In forested areas, the successional stage affects the dynamics of small mammals and especially that of *Microtus* voles, which largely disappear where grassy clear-cut areas become unsuitable habitats when afforested (Savola et al. 2013). In Sweden, the cyclicity of *Myodes* voles remained despite natural succession or land-use change from e.g. old-growth forest to clear-cuts (Ecke et al. 2002). In Finland, a high degree of agricultural landscape fragmentation is associated with increased spatial variation in *Microtus* population growth rates, as compared to unfragmented agricultural landscapes (Huitu et al. 2004). Cyclic dynamics of common vole *Microtus arvalis* populations emerged overtime on expanding meadows in reclaimed areas in The Netherlands (van Wijngaarden 1957). All these observations imply that land-use change is capable of influencing small rodent population dynamics.

Land cover changes in combination with precipitation may well be an important predictor of rodent outbreaks in agricultural systems (Stenseth et al. 2003). Also, clear-cutting has been reported as an important driver of outbreaks in deer mice *Peromyscus maniculatus* in Canada (Sullivan and Krebs 1981). Land cover changes, the spatial structure of landscape elements, the quantity and quality of food, and general habitat availability may all promote population outbreaks, but the causality and relevance of these factors still need further research.

Nevertheless, a prominent effect of land-use change seems to be the increase in agricultural or grassland areas. This effect on landscape structure may permanently induce chronic high vole densities and outbreaks (Delattre et al. 1996; Fichet-Calvet et al. 2008), as has been observed for many species all over the world. For instance, land cover change from cropped fields to interconnected hay meadows facilitated population outbreaks of water voles *Arvicola amphibius* (Halliez et al. 2015). Agricultural irrigation increased the area of grassy crops, resulting in invasions and following eruptive dynamics of common voles *Microtus arvalis* in Spain (Luque-Larena et al. 2013). In Mongolia, outbreaks of Brandt’s vole *Microtus brandti* occurred more frequently due to increases in livestock populations (Zhang et al. 2003).

In Southeast Asia, an important factor for outbreaks of rodents in agricultural landscapes, dominated by rice, is the intensity and timing of land use. In Vietnam, the rice field rat *Rattus argentiventer* causes chronic problems, but their population dynamics have changed markedly when the agricultural management shifted from two to three rice crops per year in the Mekong delta. Breeding of the rice field rat is synchronized with the pre-booting stage of rice, with more crops per year resulting in more breeding seasons (Lam 1983; Brown et al. 2005). This effect is further exacerbated if there are conditions that lead to higher asynchrony of cropping (Brown et al. 2011).

Land-use change can occur over large spatial scales (e.g. clear-cutting of boreal forest, the succession of arable land after the collapse of the Soviet Union, forest fires in North America), but they are not temporally synchronized and hardly recur with a specific time interval. Rather than inducing cyclicity *per se*, changes in land use and landscape structure may create conditions suitable for cyclic population dynamics, e.g. through changes in trophic interactions. Hence, the study of land-use change may give new insight into the dynamics of small rodent populations. In particular, the repeated observation that the prevalence of outbreaks generally increases due to more homogeneous land cover on large spatial scales may be of interest, especially for rodents that are well adapted to these modified habitats. An essential question related to land-use changes is:

6. *What are the possible pathways of how changes in land use and landscape structure affect small mammal population dynamics?*

## Community processes, conclusions and further questions

The fundamental basis for understanding small rodent population dynamics lies in its inherent annual density variation—a peak in the late breeding season, and low numbers at the end of the non-breeding season. The demographic machinery that generates this pattern is fairly well understood. If the annual density fluctuation exceeds the year-to-year variation in peak and low numbers, respectively, this hints to intrinsic regulating mechanisms, together with limiting food resources, that keep the populations within an envelope of regular density fluctuation only. However, populations can escape from these mechanisms, either occasionally as in the case of outbreaks, or following a systematic temporal pattern with a persistent sequence of the four-cycle phases. The comparison of both eruptive and cyclic populations that we follow in this review will, therefore, enable the identification of the driving force, or forces, that cause the dramatic and still enigmatic bursts in rodent numbers.

Modern ecology is based on the experimental testing of hypotheses. Thus, also population ecologists of small rodents have leaned towards single–species processes and population dynamics, as this allows simpler experimental designs. Manipulating whole communities and defining the causality of responses and population processes in different species of the community is difficult if not impossible. In the northern hemisphere, we have three main rodent genera: *Myodes*, *Microtus* and lemmings. They have three different habitat preferences, three different diets, i.e. seeds, buds, lichens for *Myodes*, graminoids for *Microtus* and mosses for lemmings (Hansson and Henttonen 1985) but see Soininen et al. (2017b), and probably three different social systems—but still, they have synchronised dynamics over large areas. Despite contrasting diets and social systems, the species are exposed to common predators and share the same abiotic factors, environmental change and climate. The community ecological approaches applied by Hansson and Henttonen (1988) and Henttonen (2000) should encourage us to develop other comparative studies on community levels (e.g. Sundell et al. 2012, Ecke et al. 2017) and even experiments monitoring concurrently the responses of several species to community-level manipulations in environmental variables, including food, predation or the social environment (Eccard and Ylönen 2007, Sundell et al. 2008, Eccard et al. 2011).

Further, we should try to understand why the dynamics of some rodent species deviate from the dynamics of other members of the rodent guild in a certain area. In particular, it may be worth searching for a temporal factor that first causes outbreaks in some species, which in turn releases other species from predation pressure so that they can start growing. A potential study system may involve the large European water vole *Arvicola amphibius* in Northern Europe, and *Apodemus* mice species and the tiny harvest mouse *Micromys minutus* in Central Europe.

Two essential questions on the community level would be:

7. *How does the temporal synchrony in the dynamics within the small rodent community shape population cycles and outbreaks?*

and

8. *How does the small rodent community affect the whole ecosystem dynamics?*

The small rodent communities are good models for these kinds of questions as they are logistically easy to work with in natural populations. There is, however, an inherent problem in studying the low phase in the wild, as it is difficult or even impossible to obtain large enough samples to reveal which of the vital population parameters are affected. Just on that account, we urgently need to intensify studies on the low phase of the cycles.

Although we discussed food resources repeatedly throughout the paper, there is still a lack of data specifying rodent diet (but see e.g. Hansson 1971; Hansson and Larsson 1978; Soininen et al. 2018) and potential shifts in the diet through an outbreak or a cycle. Many of the eruptive populations in homogenous agricultural land seem to be directly connected to ample food resources during the outbreaks. However, studying diet changes is challenging because the mechanisms may involve both the quantity and quality of food resources, and their interactions with other factors, for instance predation and/or pathogens and diseases.

An essential question for future research may be:

9. *Is there a systematic shift in small rodent diet through a population cycle or season that is important for shaping the dynamics?*

We have not discussed new insights from studies of how small rodent behaviour may affect population dynamics (Sih et al. 2012). During certain phases of the cycles, different individual behavioural strategies—now often called animal personalities—could be advantageous (Boonstra and Krebs 1979; Eccard and Herde 2013; Nicolaus et al. 2016). The concept combines different aspects such as dispersal, physiology and life history characteristics of individuals into a composite syndrome (Réale et al. 2010; Carere and Maestripieri 2013; Dammhahn et al. 2018). With cycles likely resulting from community-level interactions, a novel approach would be to look at how cycle phases affect in turn individual differences in immunological responses, survival and reproductive investment.

Most studies on population dynamics in small rodents search for one factor shaping population dynamics, possibly even confined to one particular cycle phase. Recurrently, however, we are almost inevitably faced with questions about how various factors interact. For some of these, we assume multifactorial frameworks, but hard data are largely missing. For instance, there is experimental evidence that food supply during winter increases survival (Johnsen et al. 2017), because all animals without access to supplemental food die and the population crashes. But it remains an open question whether nourishment as such is the only factor, or whether food availability secures healthy animals that can better escape predation or diseases (but see Huitu et al. 2003). This novel multifactorial approach raises the last and probably most central question:

10. *How do different factors such as seasonality, predation, behaviour, food and diseases interact?*

We probably know much more about the mechanisms causing outbreaks than about the driving forces of population cycles, even though population cycles have been under long and intensive research. Outbreaks occur as a response to a more or less stochastic pulse of resource availability. Compared to that, many more factors are suggested to be involved in cyclic dynamics (Fig. 1). Such networks of mutual interactions are complex and difficult to disentangle in practice, but seen in this light the cycles are not at all a mystic phenomenon. Cyclic vole populations and their pathogens have overall received relatively little research attention, and more research in this field is sorely needed. We already know many of the mechanisms involved in population dynamics, hence new questions of how they act together seem to be a most promising direction for a better understanding of outbreaks as well as population cycles. Obviously, the study of small rodent population dynamics will give new insight into general population ecological theory also in the future.
