# Correction to: Population cycles and outbreaks of small rodents: ten essential questions we still need to solve

**DOI:** 10.1007/s00442-021-04856-4

**Published:** 2021-01-30

**Authors:** Harry P. Andreassen, Janne Sundell, Fraucke Ecke, Stefan Halle, Marko Haapakoski, Heikki Henttonen, Otso Huitu, Jens Jacob, Kaja Johnsen, Esa Koskela, Juan Jose Luque-Larena, Nicolas Lecomte, Herwig Leirs, Joachim Mariën, Magne Neby, Osmo Rätti, Thorbjörn Sievert, Grant R. Singleton, Joannes van Cann, Bram Vanden Broecke, Hannu Ylönen

**Affiliations:** 1 Faculty of Applied Ecology, Agricultural Sciences and Biotechnology, Inland Norway University of Applied Sciences, Campus Evenstad, 2480 Koppang, Norway; 2 Lammi Biological Station, University of Helsinki, Pääjärventie 320, 16900 Lammi, Finland; 3 Department of Biological and Environmental Science, Konnevesi Research Station, University of Jyväskylä, P.O. Box 35, 40014 Jyväskylä, Finland; 4 Department of Wildlife, Fish, and Environmental Studies, Swedish University of Agricultural Sciences, Skogsmarksgränd, 90183 Umeå, Sweden; 5 Institute of Ecology and Evolution, Friedrich Schiller University Jena, Dornburger Str. 159, 07743 Jena, Germany; 6 Terrestrial Population Dynamics, Natural Resources Institute Finland, Latokartanonkaari 9, 00790 Helsinki, Finland; 7 Federal Research Centre for Cultivated Plants, Vertebrate Research, Julius Kühn-Institut, Toppheideweg 88, 48161 Münster, Germany; 8 Department of Biological and Environmental Science, University of Jyväskylä, P.O. Box 35, 40014 Jyväskylä, Finland; 9 Departamento de Ciencias Agroforestales, Escuela Tecnica Superior de Ingenierıas Agrarias, Universidad de Valladolid, Campus La Yutera, Avenida de Madrid 44, 34004 Palencia, Spain; 10 Canada Research Chair in Polar and Boreal Ecology and Centre D’Études Nordiques, Department of Biology, Université de Moncton, 18 Avenue Antonine-Maillet, Moncton, NB E1A 3E9 Canada; 11 Evolutionary Ecology Group, Department of Biology, University of Antwerp, Universiteitslain 1, 2610 Wilrijk, Belgium; 12 Arctic Centre, University of Lapland, P.O. Box 122, 96101 Rovaniemi, Finland; 13 International Rice Research Institute, DAPO Box 7777, Metro Manila, Philippines; 14 Natural Resources Institute, University of Greenwich, Chatham Marine, Kent, ME4 4TB UK

## Correction to: Oecologia 10.1007/s00442-020-04810-w

Authors would like to correct error in affiliation in the original publication of the article. Correct affiliation of Thorbjörn Sievert is updated here.

Correct affiliation of Thorbjörn Sievert to (3) not (2).

